# Selections and Abstracts

**Published:** 1916-03

**Authors:** 


### SELECTIONS AND ABSTRACTS

### U. S. DEPARTMENT OF LABOR

Children s Bureau Washington.

There are 1727 communities considering some preparation for Baby Week, according to the inquiries received by the Children's Bureau of the U. S. Department of Labor. This number does not include those of whose interest in the campaign work has come to the Bureau indirectly.

The letters about Baby Week are still coming in from every State in the Union and from every type of community, such as a Colorado settlement forty miles from a railroad, a club of women on one of the Government reclamation projects, a Montana coal mining town with a large foreign population, a southern mill village, and a club of farm women in a middle western state.

Texas has its own Baby Week slogan—Baby Health is Texas Wealth—and Mississippi has started a competition to secure a slogan for that state. North Dakota reports plans for a state-wide essay contest in the public schools. In a few state campaigns the State Federation of Women's Clubs, the State University Extension Department, the State officials, and those who are especially interested in education are all co-operating in the Baby Week campaign.

Many large cities are going to have a Baby Week. Definite plans are under way in Albany, Baltimore, Boston, Cleveland, Milwaukee, Minneapolis, Philadelphia, Richmond, San Francisco, Washington and other cities. New York had a successful Baby Week in 1914 and will probably hold another this year in the late spring.

In its suggestions for Baby Week observance the Children's Bureau lays special emphasis on the opportunity it affords for extending permanent work for infant welfare, such as infant welfare stations, visiting nursing, special nursing and instruction for prospective mothers, city inspection of milk, special work for the prevention of blindness, and little mothers' classes and home nursing instruction for school girls in the upper grades.

---

### HOW THE GOVERNMENT IS MEETING THE MALARIA PROBLEM.

Four per cent of the inhabitants of certain sections of the South have malaria. This estimate, based on the reporting of 204,881 cases during 1914, has led the United States Public

Health Service to give increased attention to the malaria problem, according to the annual report of the Surgeon General. Of 13,-526 blood specimens examined by Government officers during the year 1,797 showed malarial infection. The infection rate among white persons was above eight per cent. and among colored persons twenty per cent. In two counties in the Yazoo Valley, 40 out of every 100 inhabitants presented evidence of the disease.

Striking as the above figures are they are no more remarkable than those relating to the reduction in the incidence of the disease following surveys of the Public Health Service at 34 places in nearly every state of the South. In some instances from an incidence of fifteen per cent. in 1914, a reduction has been accomplished to less than four or five per cent, in 1915.

One of the important scientific discoveries made during the year was in regard to the continuance of the disease from season to season. Over 2000 Anopheline mosquitoes in malarious districts were dissected, during the early spring months, without finding a single infected insect, and not until May 15, 1915, was the first parasite in the body of a mosquito discovered. The Public Health Service, therefore, concludes that mosquitoes in the latitude of the Southern States ordinarily do not carry the infection through the winter. This discovery indicates that protection from malaria may be secured by treating human carriers with quinine previous to the middle of May, thus preventing any infection from chronic sufferers reaching mosquitoes and being transmitted by them to other persons.

Although quinine remains the best means of treating malaria and is also of marked benefit in preventing infection, the eradication of the disease as a whole rests upon the destruction of the breeding places of Anopheline mosquitoes. The Public Health Service, therefore, is urging a definite campaign of draining standing water, the filling of low places, and the regrading and training of streams where malarial mosquitoes breed. The oiling of breeding places, and the stocking of streams with top-feeding minnows, are further recommended. The Service also gives advice regarding screening, and other preventive measures as a part of the educational campaigns conducted in sections of infected territory.

This study is typical of the scientific investigations which are being carried on eradicating the disease. The malaria work now includes the collection of mobidity data, malaria surveys, demonstration work, scientific field and laboratory studies, educational campaigns, and special studies of impounded water and drainage projects.

---

### NEW TREATMENT FOR BURNS.*

Henry Richmond Slack, Ph.M., M. D.

In reviewing the literature of surgery one is impressed with the wonderful advances made in the last quarter of a century, This is especially true in abdominal surgery, thoracic surgery, brain surgery, bone surgery and surgery of the special organs. Burns are treated as they were over three score years ago. It was very interesting to compare the chapter on burns in the textbook which my father studied in the fifties with the one I studied in the eighties, and those of to-day. There has been practically no change.

The same classification of burns are used, Dupuytren's, with his one to six degrees, or that of first, second and third degree burns. These are to be treated as they were some seventy years ago, with carron oil, bicarbonate of soda, white lead, etc., to exclude the air. The only difference noted was that the old textbooks recommended for the shock the administration by mouth of large doses of tincture of opium, while the latter one suggest the use of morphin hypodermically.

Over twenty years ago I began using a different treatment for burns. It was so simple and efficient that I recommended it to

---

*Read before the Fourth District Medical Society, Newnan, Ga., February 15, 1915.

my confreres, but have not heretofore published any paper on the subject. I hesitated about rushing into print with this treatment, for two reasons:

1st. It was suggested to me by reading a letter from a country doctor in an old medical journal long since defunct and I thought others might have tried it and published the treatment.

2d. Doing only an office and consultation practice, I have not had as many and varied cases as I would like before reporting the results. After corresponding with Dr. John C. Murphy, of Chicago, and looking up the literature on the subject, I decided it was best not to wait any longer to state that the immediate application of tincture of ferric chlorid is the best treatment for burns.

Case 1.—Myself. Was lecturing on chemistry to a class in the Southern Female College in 1891 when in making the phosforescent sun the bell-jar broke. A globule of the ignited phosforus got under the nail of the second finger of the right hand, making a deep and painful burn. A bottle of tincture of ferric chlorid was sitting on the table with the reagents. I thrust my finger into this and was surprised and pleased to feel relief from pain in less than ten minutes. When the pain returned another application gave prompt relief. This was repeated several times during the first twenty-four hours, and once daily for the next five days, and at the end of the week my finger was well. I had suffered from a similar accident before and treated with soda and carron oil. The finger was sore for over two weeks.

Case 2.—J. L., age 33, male, white, candy-maker. A new helper placed a pan of boiling candy where the cooled should have been and he thrust both hands into the boiling liquid, making a severe burn of the second degree, covering both hands and extending to the wrists. He came immediately to my office, which was next door. Applied tincture of ferric chlorid freely to the burned surface with a camel's hair pencil. The first impression was of increased pain, but in a few minutes he smiled and asked, "Is that cocain?" His hands were wrapped in gauze, being vary careful not to break the large blisters which covered them. The tincture of iron was applied four times that day and once daily for a week. In less than two weeks the skin all peeled off like a glove, and before the twentieth day he was making candy again.

Case 3.—S. H. Pretty little girl of 18 months. Fell into a bed of hot coals and had a burn of third degree on cheek and hand. The mother was distressed for fear that the child's face would be disfigured by scars. I gave 30 minims of paregoric and applied tincture of ferric chlorid freely over the burns, and in less than 15 minutes the child was asleep. Five more applications were made during the next 24 hours and then twice daily for five days, and once daily for the next week and the child recovered without any scars.

I could report a large number of cases of first and second degree burns successfully treated with ferric chlorid, but only had this one of the third degree, and it healed without pus and left no scar.

Now, how does it act? Tincture of ferric chlorid is an alcoholic solution of $Fe^2Cl^6$. We know that ethyl alcohol is one of the best antiseptics we have and ferric chlorid one of the most powerful astringents. The alcohol renders the wound aseptic and the ferric chlorid toughens the skin and makes a dry scarf epidermis in burns of the first and second degrees, and a dry impervious coat of iron on those of the third degree.

Now, a word as to treatment. If the burn is extensive give at least twice the usual dose of morphin hypodermically. Paint the entire surface over with ferric chlorid several times with cotton applicator or camel's hair pencil, being careful not to break the blisters in second degree burns or to remove the charred surface in third degree. Then apply dry gauze dressing. The first impression is of increased pain, but continue the application and it soon ceases. Whenever it becomes severe reapply the tincture of iron.

Tincture of ferric chlorid is also a splendid treatment for frostbites, but I have had only one case on which to try it.

I claim for this treatment the following advantages:

1st. It relieves the pain more promptly than any other dressing.

2d. It forms a dry surface to which dressings do not stick.

3d. It prevents infection and therefore the formation of pus.

4th. Burns heal more quickly and there is less scar tissue.

5th. It is inexpensive and easily applied.

Tincture of ferric chlorid is as great an improvement in treating burns as antitoxin is in treating diphtheria, salvarsan in syphilis or emetin in amebic dysentery, and I soon hope to see it generally used.

LaGrange Sanatorium, LaGrange, Ga.

---

### SODIUM BICARBONATE IN HAY FEVER.—A PRELIMINARY REPORT.

By Kenneth E. Kellogg, M. D., New Britain, Conn., Fellow, New York Academy of Medicine.

My case records show a series of fifty cases of hay fever, covering a period of three years. The first patient presented a general acidosis with a mild and transient glycosuria; the second high specific gravity of urine with a marked acidity. Acting on the theory that the general condition served as a primary cause by reason of certain irritating qualities of the blood, making the mucous membranes hypersensitive, I gave both patients sodium bicarbonate in dram doses three times a day. Such a marked relief from the rhinitis symptoms followed that I felt justified in administering the same treatment to the remaining forty-eight.

Reviewing my records, I find that ninety per cent. of the patients enjoyed a marked amelioration of symptoms, and seventy per cent complete relief after a few days' treatment; the remaining ten per cent. were not as markedly benefited, although they all seemed to show some improvement.

The improvement of the local symptoms seemed to be independent of the exciting cause. Some suffered from the inhalation of cotton weed; some of rag weed; some of wild rose; others of golden rod; and a few presented precedent lesions of the nasal interior. The alkali acted nearly the same in the majority of cases regardless of local or general conditions. It appeared to have a desensitizing action upon the mucous membranes. Possibly it may also have had some influence in keeping the toxins of the pollen from becoming soluble.

In three cases I found it necessary to supplement the treatment by the administration of a nasal spray of sodium bicarbonate solution. Regarding the effect the alkali may have upon the anaphylaxis or the allergie phenomena, I am unprepared to speak. The fact remains, however, that at least marked relief was given in a goodly number of cases, and for this reason I feel justified in reporting them.

This is a preliminary report. It will be followed by a more detailed one later.

173 Main Street.

